# Structural Equation Modeling of the Effects of Family, Preschool, and Stunting on the Cognitive Development of School Children

**DOI:** 10.3389/fnut.2017.00017

**Published:** 2017-05-15

**Authors:** Oluwakemi Rachel Ajayi, Glenda Beverley Matthews, Myra Taylor, Jane Dene Kvalsvig, Leslie Davidson, Shuaib Kauchali, Claude Mellins

**Affiliations:** ^1^H1 Block, Statistics, School of Mathematics, Statistics and Computer Science, University of KwaZulu-Natal, Durban, South Africa; ^2^Discipline of Public Health Medicine, School of Nursing And Public Health Medicine, University of KwaZulu-Natal, Durban, South Africa; ^3^Department of Epidemiology and Paediatrics, Columbia University Medical Center, Mailman School of Public Health, New York, NY, USA; ^4^Department of Paediatrics and Child Health, University of KwaZulu-Natal, Durban, South Africa; ^5^Department of Psychiatry, The New York State Psychiatric Institute, Columbia University, HIV Center for Clinical and Behavioral Studies, New York, NY, USA

**Keywords:** nutrition, children’s health, cognitive development, preschool education, structural equation modelling

## Abstract

A recent study based on a sample of 1,580 children from five adjacent geographical locations in KwaZulu-Natal, South Africa, was carried out to examine the association of nutrition, family influence, preschool education, and disadvantages in geographical location with the cognitive development of school children. Data were collected on the children from 2009 to 2011 for this developmental study and included cognitive scores and information on the health and nutrition of the children. The current study analyzed the association of demographic variables (geographical location (site)), child variables (sex, preschool education and socioeconomic status), parental level of education (maternal and paternal), child’s health (HIV status and hemoglobin level) and anthropometric measures of nutritional status (height-for-age) with children’s cognitive outcomes. The hypothesis is that the nutritional status of children is a pathway through which the indirect effects of the variables of interest exert influence on their cognitive outcomes. Factor analysis based on principal components was used to create a variable based on the cognitive measures, correlations were used to examine the bivariate association between the variables of interest in the preliminary analysis and a path analysis was constructed, which was used for the disaggregation of the direct and indirect effects of the predictors for each cognitive test in a structural equation model. The results revealed that nutritional status directly predicts cognitive test scores and is a path through which other variables indirectly influence children’s cognitive outcome and development.

## Introduction

Cognitive ability in children and its development is critical to school outcomes as they advance in age (1). The evidence of the negative effects of malnutrition in children and later in life provided by the literature cannot be overemphasized (2, 3). This study seeks to find variables that may be associated with the cognitive development of children. In particular, malnutrition and lack of preschool facilities are expected to play a critical role in cognitive development. Based on the findings, remedial action is suggested in order to intervene in negative situations. The current research results and recommendations provide information to improve the learning environment of children in this region. This would eradicate under performance in early school life and failure in later years.

In this study, the cognitive development of children was examined extensively using aspects of their cognitive skills, namely, motor ability, sequencing, reasoning, and identifying patterns. These skills display how well the children are able to sort, count, and arrange in sequences and patterns, the objects available to them, even without any verbal instruction in some cases.

As a starting point, the current study anticipates that the cognitive outcomes are linked to the geographical location and environment (4, 5). The site of their residence provided a framework for possible environmental influences on a child’s development (6). In the current study, family, nutrition, preschool education, and geographical location are considered to be influential in a child’s cognitive development. This is in line with previous studies which have shown that preschool aged children are more likely to be affected by these factors (6).

Previous research identified the family as the first environment for growth and development in early childhood (6). Living in disadvantaged sites and households, where a sizable proportion of parents live in poverty or are less educated, has been linked to children’s development and is said to influence their cognitive outcomes. It is surmised that risks in such households impede children’s development through inadequate learning experiences within the household and the lack of stimulation available to the children.

The present study found that disadvantaged locations, where children are living in poverty with few or no preschool facilities, had a negative impact on the children’s cognitive development and that these effects persisted and remained statistically significant for all the cognitive outcomes in this study. This result provided additional information to the existing body of literature where it was reported that poverty and lower levels of parental education affected their children’s development (7, 8). From these findings, it could be inferred that the lack of skills and experiences at an early age of learning for children living in a disadvantaged area without quality learning centers exerts a significant influence on their cognitive outcomes and development (9). The present study investigates whether the negative effect of the site of location has a direct effect on the cognitive outcomes of children and whether the interaction of the disadvantage due to geographic location, and their health and physical growth in terms of stunting, and cognitive development through cognitive stimulation and preschool education is experienced indirectly.

In this manuscript, the cognitive development of children will be examined regarding the effect of variables that investigate the structural effects of nutritional status, on children’s cognitive development above and beyond the effects of family and location. Moreover, research found links between child care quality, and children’s school attendance, but child care quality may be more important for some children, than others (2). There is also a body of research which suggests that if children in less privileged areas have access to quality child care they achieve better outcomes than those who do not (10, 11). Other findings inferred that preschool education and the care strategy influence the growth of these children with time (4). This does not appear to provide any information on the possible intervening effects of preschool education with good nutrition on the association between the children’s family and the risks of the geographical location and the cognitive outcomes. The current study, therefore, seeks to investigate if preschool education with good nutrition is more important for some children than others, depending on the risks experienced via the family and the site of location.

Hence, considering the contextual factors around the children participating in this study, the aim of this research is to investigate the associations between specific predicting factors of the child variables, namely, socioeconomic status (SES), nutritional deficiencies, health status, and the structural interactions of preschool education attendance with the cognitive development of the children. Data obtained from children in the developmental study are used to investigate the direct and indirect effects of health, preschool attendance, parental education, family, and the risk of the geographic location, on children’s cognitive skills and development, through their nutritional status, in a structural equation model.

### Theoretical Framework

The development of intelligence and problem-solving abilities that begins during infancy is perceived to be influenced by a variety of factors that can provide an emotionally stable and stimulating environment for children to ensure optimal cognitive development. When children are not raised in such an environment or are deprived of positive experiences, learning disabilities and other cognitive delays might ensue.

Previous research has described the significance of the effects of child-related variables to be ecological, where the children interact with a system with significant effect on their development (4). A number of variables have been associated with poor nutrition, resulting in a pathway to under-development in young children. This however also leads to long-term effects later in life (12).

In 2011, research in sub-Saharan Africa found low height-for-age (an indication of stunting) with a prevalence as high as 38% (13). Several studies have highlighted the negative effects of inadequate nutritional status on different domains of development and cognition (14–16). As a result of the limited access that the children in this study have to the outside world, the variables anticipated to have an effect on their cognitive outcome are inadequate nutrition as measured by stunting, lack of preschool education, level of parental education, and geographic location.

### Family Influence and Cognitive Development

A body of research has identified the family as the first place of interaction for children, and this provides an environment for acquiring basic learning and the developmental processes (3, 17). Many studies have considered the effects of family and the risks experienced, as a pathway to explain the cognitive outcomes of children. For example, family literacy provides an indication of the parental education (18). Educated parents are more likely to provide an enriching and stimulating environment in which they engage in activities such as reading and playing with their child.

Socioeconomic status (19), maternal and paternal support (20), and the effect of depression in the home also are associated with a child’s cognitive development (21). Parents living at the poverty level are often unable to provide the same educational opportunities and material advantages compared to families in a higher income bracket. Families that live in poverty may also have disorganized homes and stressful lives which influence cognitive outcomes in their children. SES as an indication of poverty was highlighted as the most influential factor of school success, as it has proved to provide better explanation for the differences observed in children’s cognitive outcomes and development (22–25). Though SES exists in classes, the majority of the previous studies described the effect of the household income as being the most important dimension of SES. One research finding showed that the cognitive score of children from low SES families decreases during early and middle childhood (26). This study considered the household asset index to be critical in predicting the cognitive outcome of these children. The site of residence, which represents the geographical location of children, is another important factor for cognitive outcome, which appeared to be associated with behavioral problems of children between ages 3 and 12 years (27).

Researchers have reported difficulties in the interpretation of the simultaneous analysis and modeling of factors with high correlation coefficients, especially in small samples (28). The effects of the association between the family risks and cognitive outcomes in children have been directly and indirectly observed in past studies, and conceptualized to influence children’s development through more proximal factors, such as household assets (2).

The literature suggests that in order to explain the condition of children, the proximal factors from more contextual collaboration, should be studied (4).

### Preschool Education and Cognitive Development in Children

Early childhood cognitive development is a critical issue as there is rapid brain development in early childhood. To optimize this brain development, certain kinds of experiences which preschool education offers, is a benefit for cognitive development (29–31). The preschool education provides opportunity for imaginative play, and reading comprehension has been said to be rooted in the imagination. It is possible for children to comprehend whatever they are reading, only if they can imagine what the characters are doing, why they are doing it and what they might do next (32). Several intervention studies, involving children in early childhood, have shown that the use of educational programs to promote preschool skills by providing age-appropriate language, literacy and numeracy activities, can be effective as far as the targeted skills are concerned (33–36).

### Stunting and Cognitive Development in Children

Globally, 35% of the burden of disease among children aged less than 5 years and 3.5 million child deaths are as a result of stunting, severe wasting, and low birth weight (37). These provide evidence of poor nutrition which is a key factor and responsible for both maternal and child health (38–42). Though the purpose of the first millenium development goal was to reduce the number of underweight children in the under-5-year age group by 50% between 1990 and 2015, goal 1 (to eradicate extreme poverty and hunger) and goal 4 (to reduce child mortality) required improvements in child nutrition (43, 44). On an annual average at year 2014, Africa’s poverty rates continued to increase with the adverse effects of crises on food, fuel, and finances (45). This is critical to child survival, and lack of attention to poor nutrition contributed to countries failing to achieve the millennium development goals (46). In South Africa, as in many sub-Saharan African countries, the HIV/AIDS/TB epidemic is exacerbated by lack of good nutrition (47).

In South Africa as a whole, wasting (weight-for-height < −2 SD), indicative of gross malnutrition as would be found in a situation of war or famine, is not a serious public health problem, but stunting (height for age < −2 SD), which is indicative of chronic under nutrition, persists, and is detrimental to child development (48, 49).

### The Present Study

As discussed, the full development of children, especially in respect of their cognition can only be discovered as a result of the simultaneous examination of the related factors of the ecological systems where these children interact. However, the concurrent examination of the effects of the family factors, stunting, and preschool education on the cognitive outcomes of children has only been considered in a few studies (50). A number of these studies focused more on the influence that family risks exert on their development (18), while the investigation of the interaction and the effect of other factors outside the family and the cognitive development of children remained unclear. Though some studies examined the environment of interaction on children, the variables of interest in these studies are factors jointly related to both early childhood and older ages (8, 51, 52).

A previous study found that the factors, which influence children early in life, change as they grow older, due to the level of the exposure and interaction of such factors with the outside world (53–56). Hence, to bridge the gap regarding early years, the current study seeks to investigate possible factors, which are peculiar to children’s experiences in acquiring abilities for school outcomes and cognitive development. The indirect effect of family is perceived through the preschool education outcomes rather than the direct influence exerted on their cognitive development.

Hence, the current study examines the effect of family risk and preschool education in order to segregate the direct and the indirect effects on the cognitive development of children through stunting. The direct effects of preschool education on children’s cognitive outcomes, after controlling for family and stunting, are investigated. The direct effect of stunting on the cognitive outcomes of children, and the role of preschool education in compensating for family and the stunting risks, in relation to children’s cognitive development, has been of great interest to their development.

There are differences in the observed effect sizes based on the cognitive domain tested, in line with the existing literature about the importance of preschool education on the cognitive outcomes of children, especially for children from less privileged sites of residence.

With the multiple risks, which affect the cognitive development of children in this region, the current study will simultaneously investigate family factors, preschool education, and stunting on their cognitive development, as well as the mediating role of their nutritional status due to the high risk of poor nutrition, by answering the following research questions:

*Research question 1*: What is the level of interaction between the family risks, preschool attendance, nutritional status, and other variables on the cognitive development of children? The hypothesis is that children in families with higher risks and living in more disadvantaged areas are more likely to achieve low levels of cognitive skills.
*Research question 2*: To what extent does nutritional status mediate the cognitive outcomes of the children? The hypothesis is that nutritional status predicts the children’s cognitive outcomes. The path diagrams for the hypotheses will appear in the Section “Methods.”

## Methods

This study used the data set from the survey of a larger study, which was conducted between 2009 and 2011 in a rural area of KwaZulu-Natal, South Africa, for the evaluation of children’s development, which encompassed different domains of cognitive skills of the participating children. Among other characteristics, the survey collected data on child demographic factors (child’s age measured in months, sex), family factors (paternal level of education, maternal level of education), socioeconomic factors (household asset’s index, site of residence), preschool education (whether or not the child had preschool education), child health factors (child’s hemoglobin level and child’s HIV status), and on the anthropometric variable (height-for-age (hazscore)—an indication of stunting), which provide outcome measures of nutritional status. The construction and standardization of the cognitive tests used is age-appropriate and relatively culture-related (46). These were the Grover-Counter and the Kauffman’s KABC-11 subtests (Atlantis and Hand movement tests) for a total sample of 1,586 children, who were within the ages of 6–8 years.

### Sample Used for the Analysis

Although 1,586 children were invited to participate in the study, only 1,383 valid cases were used with measurements on the cognitive test outcomes and the anthropometric scores. Trained research assistants in five adjacent regions of KwaZulu-Natal collected the data from 1,582 children and caregivers, who were initially enrolled for the interview. The five local areas are characterized by different governing authorities and differences in terrain. Children who were unable to perform the tests (get tired quickly or who were not well on the day of the testing) and for whom there were no cognitive test results were excluded from the analysis.

Ethical approval was obtained for all study procedures and interview material from the University of KwaZulu-Natal Biomedical Research Ethics Committee (BF036/07) and the Columbia University Institutional Review Board. Written informed consent was obtained from parent/caregivers.

### Descriptive Statistics of Variables Associated with the Participating Children

The cognitive scores of the children vary according to each of the domain examined. Children’s scores are provided in the order of the Atlantis, Grover-Counter, and Hand movement tests with mean and SD (46.81, 16.79), (44.30, 18.61), and (6.73, 2.47), respectively. The other categorical characteristics of the data obtained for the children who participated in the current study are described in Table 1. Children’s mean age was 82.82 months with a 7.03 SD, and approximately half of the children were girls (49.6%). The percentages of the children participating from these five adjacent geographic sites were 17.4, 16.0, 10.8, 33.3, and 10.8% for sites 1–5, respectively. Of the children, 67.4% had preschool education and 3.9% had a positive HIV status. Using quintile subdivisions of the SES indices ranging from the lowest through the highest, close to half (43.4%) of the participating children had a SES below the middle class. Almost all the parents had a level of education between none and matric (grade 12), while a few reached tertiary level (0.2% and 0.1% of the mothers and fathers, respectively).

**Table 1:** **Descriptive statistics of the children’s categorical variables (*n* = 1,586)**.

Site			Sex			Preschool education			Child HIV		
	*n*	%		*n*	%		*n*	%	*n*	%
1	241	17.4	M	698	50.4	None	481	35.3	Positive	62	3.9
2	222	16.0	F	688	49.6	Received	883	64.7	Negative	1278	80.8
3	150	10.8							Unknown	241	15.2
4	461	33.3									
5	312	10.8									

**Maternal education level**			**Paternal education level**			**Socioeconomic status (SES)**					
	***n***	**%**		***n***	**%**		***n***	**%**			

0. (None)	65	4.1	0. (None)	88	5.6	1. Lowest 20%	327	20.8			
1. Grade 1–7 (primary)	223	14.1	1. Grade 1–7 (primary)	170	10.7	2. Low middle	355	22.6			
2. Grade 8–11 (high school)	665	42.0	2. Grade 8–11 (high school)	407	25.7	3. Middle	250	15.9			
3. Grade 12 (matric)	359	22.7	3. Grade 12 (matric)	437	27.6	4. High middle	314	20.0			
4. >Grade 12 (tertiary)	3	0.2	4. >Grade 12 (tertiary)	2	0.1	5. Top 20%	326	20.7			
5. Unknown	267	16.9	5. Unknown	478	30.2						

## Measures

### Dependent Variable

#### Cognitive Outcomes

In this study, all tests were conducted by mid-level trained research assistants, who directly observed and recorded scores for each of the children’s skills for motor ability, memory, and reasoning. Hand movements tested children’s ability to precisely copy a sequence of taps and children demonstrated their learning skills through their ability to accurately identify objects and their nomenclature (57, 58). The scores were standardized to reflect the appropriate normalized cognitive outcomes of every child as would be expected of other children within this age bracket in the population.

### Independent Variables

#### Sex

Child’s sex was coded as *1* = *male (reference category) and 0* = *female*.

#### Site

The children’s site of residence at the time of the study was coded *1–5 (where 5 is the reference category)*.

#### Preschool Education

There were two categories of preschool education for the children [*1* = *those who received preschool education (reference category) and 2* = *those who did not*].

#### Socioeconomic Index

The SES was measured by the household wealth asset; a quintile index with coding [1 = lowest 20%, *2* = *low middle, 3* = *middle, 4* = *high middle, and 5* = *top 20% (reference category)*].

#### Hemoglobin

The hemoglobin level in the blood of the children who participated in this study was measured using a Hemocue.

#### Parental Education

This was included in the hypothesized model as a separate measure of the level of education completed by the fathers and mothers at the time of investigation and it was coded as (*0* = *none, 1* = *attended primary school, 2* = *attended high school, 3* = *completed grade 12 (matric), 4* = *college, and 5* = *unknown)*.

#### HIV Status

During the study procedures, all caregivers were asked for their HIV test results and that of their children, and testing was offered in any case where this information could not be provided. This was categorized as *[HIV positive, HIV negative and those with an unknown status (reference category)]*.

#### Anthropometry

Children’s height was directly measured with the use of a fixed stadiometer, and the recorded height scores were normalized to *z*-scores as an indication of the mean of their nutritional status which is referred to as height-for-age *z*-score (Hazscore).

#### Mediator

The height-for-age *z*-score (Hazscore) was used as the mediating variable in the mediation model.

## Data Analysis

### Preliminary Analysis

General linear models were fitted for the examination of the association and group differences between each cognitive score and the independent variables. A factor analysis, which was based on three cognitive scores, was carried out and a principal component analysis with Eigen values greater than one was used to determine the number of principal components. This was conducted in IBM SPSS version 24 to confirm loadings of the predicting variables on each factor (59, 60). The correlation coefficient of at least.3 (i.e., ≥0.3) in the matrix made the data set suitable for factor analysis. To reduce the subjectivity of the interpretation on factors, predictors with factor loadings greater than 0.50 on a factor were considered.

### Path Analysis in the Direct Model

**Definition 1:** The total effect consists of all the coefficients of the associated variables of interest. This can be disaggregated into the direct and the indirect effects, which can be expressed as in Eq. 1 below.

(1) Total effects=Direct effects+Indirect effects

**Definition 2:** The direct effects are the coefficients on which the cognitive outcomes under investigation directly depend and are indicated by single headed arrows, originating from an independent variable to the dependent variable.

**Definition 3:** The indirect effects are the coefficients on which the cognitive outcomes indirectly depend through another intermediary effect, i.e., the mediator is directly associated with such effects and in turn directly associated with the cognitive outcomes.

Indirect effects are indicated by single headed arrows directly to the mediator and from the mediator directly to the outcomes under consideration. Hence, such an effect is said to have an indirect effect through the mediator (nutritional status) on the cognitive outcomes.

The values on a two-headed arrow in each model represents an estimate of the covariance between the two connected variables. The value on a single-headed arrow emanating from the predicting variable pointing to the dependent variable (cognitive scores) denotes the regression weight and the two other values in the order of (a, b) at the top of the boxes indicate the variance and the standard error of the estimates, respectively.

### Structural Equation Modeling

Structural equation modeling was employed to examine the association between the cognitive score, the nutritional status as a mediator in the model, and all other predicting variables, for the explanation of the direct and indirect effects on the cognitive outcomes and to examine how background characteristics through mediation directly and indirectly influenced each of the cognitive scores. To test the direct and the indirect effects of the background variables and the mediation, for the first and second hypothesis, path analysis models were constructed, and the standardized coefficients for the continuous outcome for the cognitive skills were considered as effect sizes.

Careful consideration was given to the following criteria of the model fit indices suggested by Hooper, Coughlan and Mullen (61).

- The *p*-value should be less than 0.05.
- The root mean square error of approximation (RMSEA) less than.06 indicates an adequate model fit.
- Incremental fit index (IFI) value, where the model with a value close to zero is considered as a worse model and was used to compare the estimated model to the null model.
- Akaike information criterion (AIC) value was used for the comparison of the estimated models for goodness of fit of the three cognitive scores to identify the best model.

It is worth noting that the value of the RMSEA for all models in this study was less than 0.158, hence the computation of the Comparative Fit Index was not considered, as it is highly sensitive to sample size.

## Results

A factor analysis based on three cognitive scores (Atlantis, Grover, and Hand movement) was performed to create one variable for cognitive score [A principal component analysis extracted one component with Eigen value exceeding one (1), explaining approximately 55% of the variance]. The one component solution had loadings 0.700, 0.755, and 0.763 for Atlantis, Grover, and Hand movement, respectively.

A linear regression model using the factor scores based on the three tests was run with the independent variables of interest. The results appear in Table 2.

**Table 2:** **Parameter estimates, standard errors, *t*-values, and *p*-value for the factor based on three scores**.

	Factor based on three scores			
Parameter	$\hat{\beta }$	SE	*t*	*p*-Value
Intercept	−3.597	0.426	−8.437	0.000
**Site (Ref = Site 5)**				
Site 1	0.103	0.085	−1.217	0.224
Site 2	−0.044	0.085	−0.520	0.603
Site 3	−0.172	0.099	−1.748	0.081
Site 4	−0.061	0.072	−0.840	0.401
**Gender (Ref = male)**				
Female	−0.060	0.051	−1.186	0.236
**Pre–school education (Ref = received)**
None	−0.156	0.060	−2.606	0.009
**HIV Status (Ref = unknown)**
HIV positive	−0.070	0.152	−0.462	0.644
HIV negative	0.091	0.075	1.215	0.225
Age (months)	0.046	0.004	11.500	0.000
Height-for-age *z*-score	0.157	0.027	5.865	0.000
Hemoglobin	−0.001	0.022	−0.047	0.962
**SES (Ref = 5)**
SES 1	−0.014	0.080	−0.180	0.857
SES 2	−0.008	0.078	−0.099	0.921
SES 3	0.096	0.085	1.131	0.258
SES 4	−0.016	0.078	−0.198	0.843
**Maternal education (Ref = unknown)**
Maternal education = 0	−0.084	0.142	−0.589	0.556
Maternal education = 1	−0.236	0.097	−2.438	0.015
Maternal education = 2	−0.097	0.076	−1.281	0.201
Maternal education = 3	0.121	0.085	1.434	0.152
Maternal education = 4	0.050	0.653	0.076	0.939
**Paternal education (Ref = unknown)**
Paternal education = 0	−0.010	0.121	−0.079	0.937
Paternal education = 1	−0.028	0.092	−0.303	0.762
Paternal education = 2	0.081	0.068	1.184	0.237
Paternal education = 3	0.127	0.068	1.854	0.064

Preschool education, age, height-for-age and mothers’ education were significantly associated with the cognitive score variable. The remaining variables, which included site, gender, HIV status, SES, fathers’ education, and hemoglobin level, were not significant.

In a multivariate analysis using the three cognitive scores, the *F*- and *p*-values are as follows: preschool education (*F*-value = 6.791, *p*-value = 0.009), mother’s education (*F*-value = 4.414, *p*-value = 0.02), height-for-age *z*-score (*F*-value = 34.393, *p*-value = 0.000), and age (in months) (*F*-value = 132.240, *p*-value = 0.000).

### Correlational Analysis of the Variables of Interest

The bivariate correlations between each of the variables included in the analysis are provided in Table 3 below.

**Table 3:** **Bivariate correlations**.

	1	2	3	4	5	6
1. Atlantis	1					
2. Grover	0.378**	1				
3. Hand movement	0.336**	0.435**	1			
4. Age (month)	0.312**	0.442**	0.368**	1		
5. Height-for-age	0.151**	0.182**	0.135**	0.028	1	
6. Hemoglobin	−0.005	0.040	0.054*	−0.002	0.111**	1

*p < 0.05.

**p < 0.01.

Age was positively correlated with all the cognitive skills (Atlantis, Grover, and Hand Movement tests), Height-for-age *z*-score (the mediator) had a positive correlation with all the test scores and hemoglobin was positively correlated with hand movement test score and height-for-age but had a negative correlation with Atlantis test score and age (in months).

### Path Analysis in the Mediation Model

**Figure 1:**
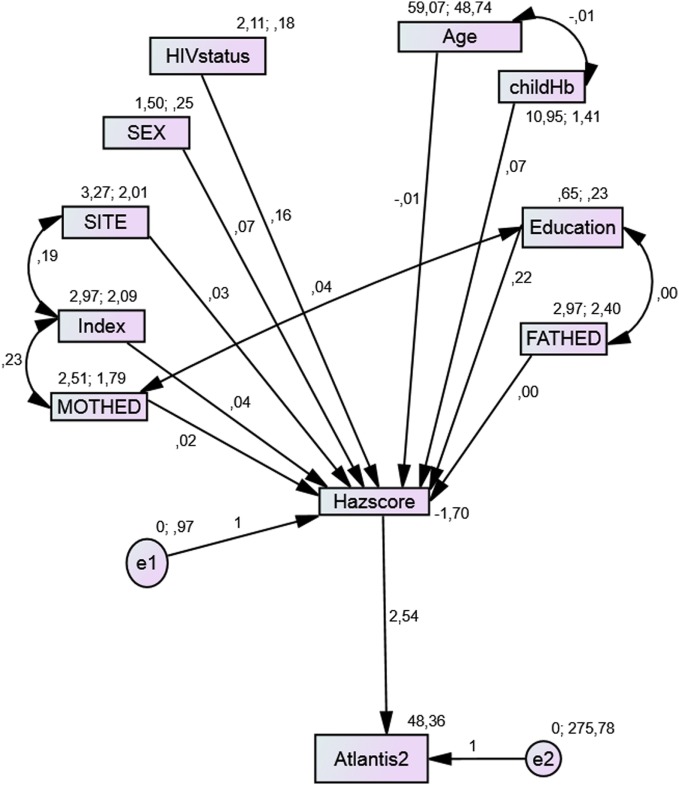
**Model with the mediation of nutritional status for Atlantis test**.

**Figure 2:**
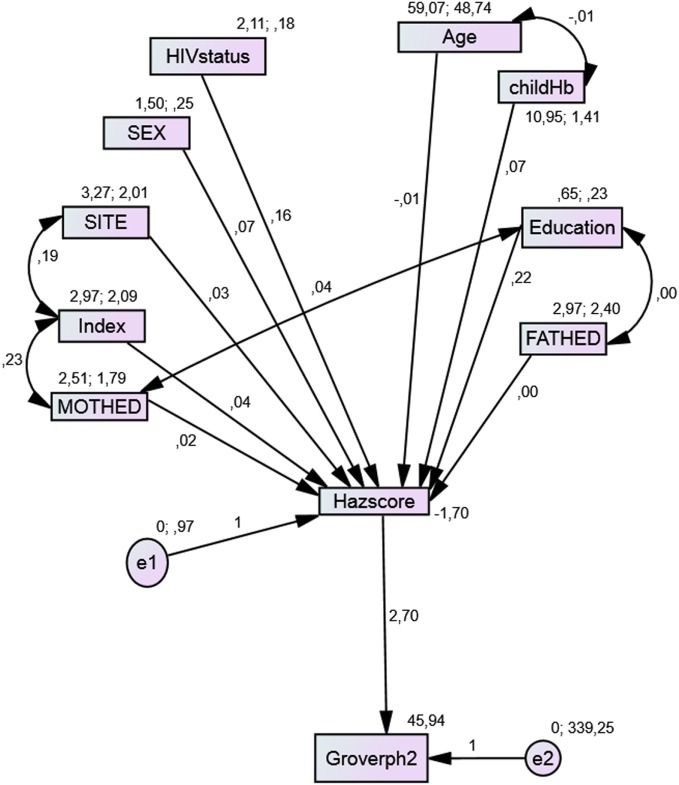
**Model with the mediation of nutritional status for Grover test**.

**Figure 3:**
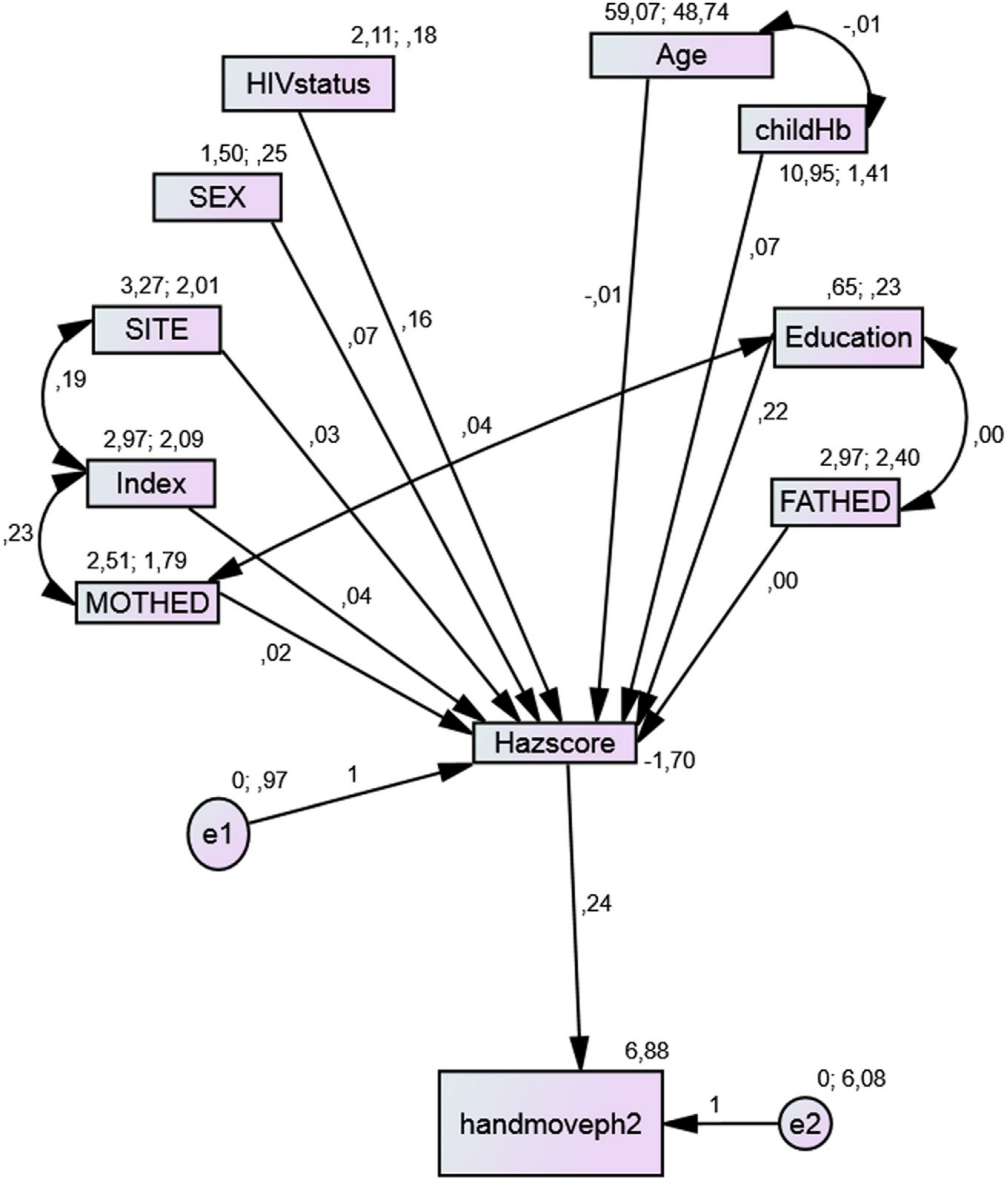
**Model with the mediation of nutritional status for Hand movement**.

From the path diagrams of the cognitive outcomes, each model provided a good fit with the data and provides a better model when compared with the linear regression model of the same outcome. Though the initial models did not fit the data well, children who had preschool education as well as those with good height-for-age *z*-scores had better cognitive test scores. Site, mothers’ level of education, and SES were related indirectly to these test scores through nutritional status. Children residing in sites with better resources, whose mothers had higher levels of education, with higher levels of SES index, were associated with both higher nutritional status and better scores in each test (Figures 1–3). While the indirect effects of other variables were not statistically significant.

## Model Fit

### Atlantis Test

The Atlantis test model (Figure 1) had a good fit with an AIC less than the independence model, an IFI that is significantly different from the null model, and RMSEA < 0.08.

### Grover-Counter Test

The model for the Grover-Counter test (Figure 2) had a good fit with AIC less than the null model, IFI different from the independent model, and RMSEA < 0.08.

### Hand Movement Test

Scores obtained for the Hand movement test (Figure 3) also fitted the model well with AIC, IFI, and RMSEA < 0.08.

The results, which provided support for the two hypotheses tested with the mediation model, are outlined in Table 4. With all the variables of interest included in the model analysis, mothers’ level of education, socioeconomic index, site, HIV status, and preschool education were significant and the model fitted the data well (Figures 1–3).

**Table 4:** **Maximum likelihood estimates of covariance in the model**.

Covariance	Correlation estimate	Standard error	Covariance estimate	*p*-Value
Mothed ↔ Index (SES)	0.225	0.049	4.602	***
Site ↔ Index (SES)	0.191	0.055	3.470	***
Age ↔ childHb	0.009	0.223	0.042	0.966
Hazscore ↔ HIV status	0.034	0.011	3.007	0.003
Hazscore ↔ Age	−0.198	0.188	−1.052	0.293
Education ↔ Fathed	−0.006	0.020	−0.281	0.779
Education ↔ Mothed	0.045	0.017	2.635	0.008
Hazscore ↔ Index (SES)	0.106	0.039	2.744	0.006
Hazscore ↔ Site	0.043	0.038	1.130	0.258

***p < 0.001.

## Discussion

The cognitive development of children is a complex topic. This study examines the multivariable effects of preschool attendance, the influence of family, nutritional status as determined by height-for-age *z*-score and the geographical location on cognitive outcomes of children in an impoverished region of KwaZulu-Natal. A path diagram for the direct and indirect effects of variables on cognitive scores was constructed. The hypothesis is that certain variables affect the nutritional status of a child and this, in turn, has a direct effect on the cognitive outcome of the child. Nutrition is viewed as a mediator in the association with the cognitive scores. All analyses from which the unbiased estimates and the testing of hypotheses were done with Version 24 of IBM SPSS and the add-on package (Amos).

### Nutrition and Its Mediating Effects on Children’s Outcome

The first hypothesis addresses the direct effects of the independent variables on child outcomes. Generally, in the current study, the major risk factors associated with poor nutritional status of children were the rural site where the child lived and lack of preschool education. This study observed that the effect of sex on any of the cognitive outcomes was not significant. To further investigate the direct effects of the variables of interest (examined in the first hypothesis testing), the second hypothesis was tested for the indirect effects of these same variables on child outcomes through the nutritional outcomes (height-for-age *z*-score), using the mediation model in which nutritional status was a mediator for the indirect effects of site and family, simultaneously with preschool education, on the cognitive outcomes.

The mediating effects of height-for-age *z*-score through the associations of the independent variables of interest and the children’s cognitive outcomes clearly provide support for the hypothesis of the mediation model. It is evident that in all three tests of cognition, children who had the lowest scores were generally those who also had low height-for-age scores. Apparently, high cognitive scores were predicted by good nutritional status at an early age in these children. The negative effects of such conditions could persist and extend beyond these age groups unless adequate measures of intervention are promptly implemented. All the children and their caregivers were brought to the research clinic and provided with refreshments prior to the children being tested and afterward.

In the mediating mechanism, the nutritional status in turn influenced the outcomes of these children on the tests of cognition. Further, the statistical significance level, and the effect sizes differed by specific child outcome. In contrast with a report from a previous study, where SES predicted both nutritional status and children’s outcomes (62, 63), the association of the socioeconomic risk index was strongly supported in the mediation model for all the children’s cognitive scores, but the direct effect was not significant in the current study. The reason could be that the children who are socioeconomically disadvantaged, lack resources, which could be helpful to facilitate their learning opportunities.

Exposure to risk factors in the family has been associated with young children’s school failure (64–66), and inadequate resources for the children, may in turn, impact on their cognitive outcomes and development. In line with the present findings, low family SES and low levels of parental educational attainment were said to result in less social and economic resources for young children and higher levels of intellectual and social and emotional problems (67), thus reducing the likelihood that children will attain full cognitive development. However, some studies have found both SES and the level of education not to be significantly associated with children’s cognitive development. This latter research suggested that it is possible that family socioeconomic risk factors indirectly affect children’s development through other risk factors such as parental education and health (65). Parents with low levels of family SES may not have enough resources and opportunities to provide learning materials, role modeling, or cognitive stimulating support, due to health conditions, which put the child at risk (68). From our findings, family socioeconomic disadvantage may restrict parents’ emotional well-being or increase the possibility of depressive symptoms as reported in previous papers (69).

The family interaction may reflect pressure and stress due to the lack of necessary amenities, which could be unhealthy and in turn, could hinder children from developing optimally (8).

Although the home context has in many studies found to be an important factor in children’s early development (70), few studies have investigated the cognitive outcome of children being influenced by geographic location. In contrast, a number of studies have found an effect of the geographic location and inadequate resources on children’s cognitive development, at older ages (70). Since these children have their first learning experiences in the home with limited opportunities of interacting with the outside world at this early stage, schools and learning facilities later serve as the route through which children overcome the barriers in the process of their cognitive development (70).

Nowadays since gender equality has promoted the chances of employment opportunities for mothers, more children have to be at a place of care, which may be quite different from their households. The significant difference, observed between those children who attend preschool and those who do not, provided statistical evidence that children attending a preschool facility of some kind have a better chance of learning and acquiring cognitive skills for their development. There is also a higher likelihood that these children have better cognitive outcomes in later school years (58, 66). This highlighted the importance of all children receiving preschool education which requires intervention so that the negative effects of inadequate or unavailability of facilities for preschool education are addressed, in order to improve children’s cognitive outcomes and development for school success. Previous studies have sought to identify and evaluate strategies that could provide good care for children (8, 9, 63), but few studies have examined the significant effects of such care on their cognitive outcomes. The current research has identified the importance of preschool education not only for child care, but also for improvement of their cognitive skills and development for optimal school success. The findings of this study highlight the importance for local authorities to provide preschool facilities in the rural communities.

There are a range of policies in South Africa that aim to address the needs of children (71) but as our study shows many children’s requirements remain unmet, with a prevalence of stunting of 27% in children under 5 years of age (72). The South African Government provides impoverished caregivers of every child up to 18 years with a child support grant (csg) of R350/month and free health care (73), but access requires one to have an identity book for the CSG, and to reach a primary health care clinic for services. These are requirements that poor households may not be able to meet, due to lack of resources to cover transport and other costs, and highlights the impact of the geographic area where the children in this study lived. The lack of household food security in 14.8% of KwaZulu-Natal homes (74) and the inadequacy of children’s diets, is exacerbated by the high rate of infectious diseases. Although provision of antiretroviral medication is free, there remains the cost of monthly clinic visits, since clinics are few and often far away. South Africa has made progress in the control of HIV and reducing maternal and child mortality and morbidity.

The underlying causes of stunting (low height-for-age) are poverty, poor maternal health during pregnancy, household food insecurity, inadequate access to maternal, and child health services, as well as lack of access to adequate water and sanitation (72) and the latter promotes diarrheal diseases and soil-transmitted helminth infections. Studies have shown that children, who are chronically under-nourished in their early years of life, fail to thrive, and achieve their full potential, as the first 2 years of life are critical for children. Furthermore, children require a diet that includes the essential micronutrients such as vitamin A and iron, but in poor homes, there is often a lack of vitamins and minerals (75).

Current policy also requires every child to attend a preschool grade R class, prior to formal schooling and this is being increasingly achieved (76) but the quality of the early childhood education that is provided, has yet to be evaluated.

## Conclusion

This study was conducted to identify the risk factors associated with the cognitive outcomes of children and the association between the health risks, malnutrition, disadvantages resulting from the site of residence, whether or not the child had received preschool education, socioeconomic risk, parental level of education, and children’s skills and development. In the current study, a preschool education experience strongly predicts higher cognitive outcomes. This study considered it important to find the pathways as to how the contexts surrounding children influence their cognitive outcomes and development in early childhood. This offers opportunities for intervention and prevention of poor cognitive outcomes, which contribute to children’s school failure as suggested by Bronfenbrenner (6). The primary contexts considered in the prediction of children’s cognitive outcomes and development were nutrition, preschool education, and family influence. Following the results of these findings, this study examined the pathways through which nutritional status mediated children’s cognitive outcomes.

The cognitive scores of these children were directly predicted by preschool education, nutrition (height-for-age), but the direct effects of SES and maternal level of education were not statistically significant. Good nutritional status mediated the association between the socioeconomic risk index and children’s cognitive skills. The effect of socioeconomic risk, mothers’ level of education, and site of residence were indirectly observed to be influencing children’s cognitive outcome with statistical significance in the mediation model.

The present study found that good nutrition mediated children’s cognitive development and serves as a compensation for the negative effect of other risks in relation to cognitive skills. The development of children’s academic outcomes is a national policy goal. The strong predictors identified in this study include health, nutrition, and preschool facilities. This study emphasizes the importance of addressing these concerns in order to change the developmental trajectory of millions of disadvantaged children. The results indicate that it would make a significant difference if children’s development was prioritized and received the required investment.

## Ethics Statement

The ethical clearance for this study was given by the Biomedical Research Ethics’ Committee of the University of KwaZulu-Natal, South Africa (BF036/07) and Columbia University, USA.

## Author Contributions

OA (corresponding author), GM (co-author), MT (co-author), JK (co-author), LD (co-author), SK (co-author), and CM (co-author) have made substantial contributions to the conception or design of the work; or the acquisition, analysis, or interpretation of data for the work.

## Conflict of Interest Statement

The authors declare that the research was conducted in the absence of any commercial or financial relationships that could be construed as a potential conflict of interest.

## References

[1] Haskins R Beyond metaphor: the efficacy of early childhood education. Am Psychol (1989) 44:274–82. 10.1037/0003-066X.44.2.274

[2] Peisner-Feinberg ES Burchinal MR Clifford RM Culkin ML Howes C Keagan SL The relation of preschool child-care quality to children’s cognitive and social developmental trajectories through second grade. Child Dev (2001) 72(5):1534–53. 10.1111/1467-8624.00364 11699686

[3] Shores RE Wehby JH Analyzing the classroom social behavior of students with EBD. J Emotional Behav Disord (1999) 7(4):194–9. 10.1177/106342669900700401

[4] Bronfenbrenner U The experimental ecology of education. Educ Res (1976) 5(9):5–15. 10.2307/1174755

[5] Bronfenbrenner U Ecology of the family as a context for human development: research perspectives. Dev Psychol (1986) 22(6):723–42. 10.1037/0012-1649.22.6.723

[6] Bronfenbrenner U Ecological models of human development. 2nd ed In: Gauvain M Cole M, editors. Readings on the Development of Children. Oxford, New York: Elsevier, Freeman (1994). p. 37–43.

[7] Dupere V Leventhal T Crosnoe R Dion E Understanding the positive role of neighbourhood socioeconomic advantage in achievement: the contribution of the home, child care, and school environments. Dev Psychol (2010) 46(5):1227–44. 10.1037/a0020211 20822235 PMC3100662

[8] Leventhal T Brooks-Gunn J The neighbourhoods they live in: the effects of neighborhood residence on child and adolescent. Psychol Bull (2000) 126(2):309 10.1037/0033-2909.126.2.309 10748645

[9] Kohen DE Leventhal T Dahinten VS McIntosh CN. Neighborhood disadvantage: pathways of effects for young children. Child Dev (2008) 79(1):156–69. 10.1111/j.1467-8624.2007.01117.x 18269515

[10] Votruba-Drzal E Coley LR Chase-Lansdale LP Child care and low-income children’s development: direct and moderated effects. Child Dev (2004) 75(1):296–312. 10.1111/j.1467-8624.2004.00670.x 15015691

[11] Pollitt E Golub M Gorman K Grantham-McGregor SM Levitsky D Schürch B A reconceptualization of the effects of undernutrition on children’s biological, psychosocial, and behavioral development. Social Policy Report. (1996).

[12] De Onis M Blössner M Borghi E Prevalence and trends of stunting among pre-school children, 1990-2020. Public Health Nutr (2011) 15(1):142–8. 10.1017/S1368980011001315 21752311

[13] Duc LT The Effect of Early Age Stunting on Cognitive Achievement among Children in Vietnam: Young Lives. Oxford: Young Lives (2009).

[14] Chang-Lopez SM Effects of Early Childhood Stunting on Behaviour, School Achievement and Fine Motor Abilities at Age 11-12 Years. St. Augustine: The University of the West Indies (2007).

[15] Pollitt E Husaini MA Harahap H Halati S Nugraheni A Otto S Stunting and delayed motor development in rural West Java. Am J Human Biol (1994) 6:627–35. 10.1002/ajhb.1310060511 28548341

[16] Callender KA Olson SL Choe DE Sameroff AJ. The effects of parental depressive symptoms, appraisals, and physical punishment on later child externalizing behavior. J Abnorm Child Psychol (2012) 40(3):471–83. 10.1007/s10802-011-9572-9 21947616 PMC4100716

[17] Griffin EA Morrison FJ The unique contribution of home literacy environment to differences in early literacy skills. Early Child Dev Care (1997) 12(7–128):233–43. 10.1080/0300443971270119

[18] Brooks-Gunn J Duncan GJ The effects of poverty on children. Future Child (1997) 7(2):55–71. 10.2307/1602387 9299837

[19] Pachter LM Auinger P Palmer R Weitzman M. Do parenting and the home environment, maternal depression, neighborhood, and chronic poverty affect child behavioral problems differently in different racial-ethnic groups? Pediatrics (2006) 117(4):1329–38. 10.1542/peds.2005-1784 16585331 PMC1475725

[20] Cummings EM Davies PT Maternal depression and child development. J Child Psychol Psychiatry (1994) 35(1):73–112. 10.1111/j.1469-7610.1994.tb01133.x 8163630

[21] Bracken SS Fischel JE Family reading behavior and early literacy skills in preschool children from low-income backgrounds. Early Educ Dev (2008) 19(1):45–67. 10.1080/10409280701838835

[22] Egeland B Kalkoske M Gottesman N Erickson MF. Preschool behavior problems: stability and factors accounting for change. J Child Psychol Psychiatry (1990) 31(6):891–909. 10.1111/j.1469-7610.1990.tb00832.x 2246340

[23] Biederman J Milberger S Faraone SV Kiely K Guite J Mick E Family-environment risk factors for attention-deficit hyperactivity disorder. A test of Rutter’s indicators of adversity. Arch General Psychiatry (1995) 52(6):464–70. 10.1001/archpsyc.1995.03950180050007 7771916

[24] Eamon MK The effects of poverty on children’s socioemotional development: an ecological systems analysis. Soc Work (2001) 46(3):256–66. 10.1093/sw/46.3.256 11495370

[25] Burchinal MR Campbell FA Bryant DM Wasik BH Ramey CT Early intervention and mediating processes in cognitive performance of children of low-income. Child Dev (1997) 68(5):935 10.2307/1132043 29106720

[26] Hofferth SL. Residential father family type and child well-being: investment versus selection. Demography (2006) 43(1):53–77. 10.1353/dem.2006.0006 16579208

[27] Burchinal MR Roberts JE Riggins JR Zeisel SA Neebe E Bryant D. Relating quality of center-based child care to early cognitive and language development longitudinally. Child Dev (2000) 71(2):339–57. 10.1111/1467-8624.00149 10834469

[28] Howes C Burchinal M Pianta R Bryant D Early D Clifford R Ready to learn? Children’s pre-academic achievement in pre-kindergarten programs. Early Child Res Q (2008) 23(1):27–50. 10.1016/j.ecresq.2008.08.001

[29] Pianta R Howes C Burchinal M Bryant D Clifford R Early D Features of pre-kindergarten programs, classrooms, and teachers: do they predict observed classroom quality and child-teacher interactions? Appl Dev Mental Sci (2005) 9:144–59. 10.1207/s1532480xads0903_2

[30] Thomason AC La Paro KM Measuring the quality of teacher–child interactions in toddler child care. Early Educ Dev (2009) 20:285–304. 10.1080/10409280902773351

[31] Teacher Support Force. Teaching children on reading and mathematics. Education for All Global Monitoring Report (2011).

[32] Bierman KL Nix RL Greenberg MT Blair C Domitrovich CE. Executive functions and school readiness intervention: impact, moderation, and mediation in the Head Start REDI program. Dev Psychopathol (2008) 20:821–43. 10.1017/S0954579408000394 18606033 PMC3205459

[33] Lonigan CJ Farver JM Philips BM Clancy-Menchetti J Promoting the development of preschool children’s emergent literacy skill: a randomized evaluation of a literacy-focused curriculum and two professional development models. Reading Writing (2011) 24:305–37. 10.1007/s11145-009-9214-6

[34] Clements DH Sarama J Effects of preschool mathematics curriculum: summative research in the Building Blocks Project. J Res Math Educ (2007) 38:136–63.

[35] Dickinson DK Caswell L Building support for language and early literacy in preschool classrooms through in-service professional development: effects of the literacy environment enrichment program (LEEP). Early Child Res Q (2007) 22:243–60. 10.1016/j.ecresq.2007.03.001

[36] Black RE Allen LH Bhutta ZA Caulfield LE de Onis M Ezzati M Maternal and child undernutrition: global and regional exposures and health consequences. Lancet (2008) 371(9608):243–60. 10.1016/S0140-6736(07)61690-0 18207566

[37] Ezzati M Lopez AD Rodgers D Murray CJL Comparative Quantification of Health Risks: Global and Regional Burden of Disease Attributable to Selected Major Risk Factors. Geneva: World Health Organization (2004). p. 163–209.

[38] Ezzati M Lopez AD Rodgers D Murray CJL Comparative Quantification of Health Risks: Global and Regional Burden of Disease Attributable to Selected Major Risk Factors. Geneva: World Health Organization (2004). p. 39–161.

[39] Pelletier DL Frongillo EA Jr. Habicht JP. Epidemiologic evidence for a potentiating effect of malnutrition on child mortality. Am J Public Health (1993) 83:1130–3. 10.2105/AJPH.83.8.1130 8342721 PMC1695164

[40] Caulfield LE De Onis M Blossner M Black RE Undernutrition as an underlying cause of child deaths associated with diarrhoea, pneumonia, malaria, and measles. Am J Clin Nutr (2004) 80:193–8. 15213048 10.1093/ajcn/80.1.193

[41] The World Bank. Repositioning Nutrition as Central to Development. A Strategy for Large-Scale Action. Washington DC: The World Bank (2006).

[42] MDG Report. Assessing Progress in Africa towards the Millennium Development Goals. Analysis of the Common African Position on the Post-2015 Development Agenda. New York, NY: Addis Ababa (2014).

[43] RSA/UNDP. The Millennium Development Goals. Stat, SA: MDG Secretariat (2010).

[44] Statistics South Africa. Mid-Year Estimates. Pretoria: Stat SA (2010).

[45] WHO. Malnutrition-The Global Picture. World Health Organization (2013). Available from: http://www.who.int/en/

[46] Ajayi OR Matthews G Taylor M Kvalsvig J Davidson LL Kauchali S Factors associated with the health and cognition of 6-year-old to 8-year-old children in KwaZulu-Natal, South Africa. Trop Med Int Health (2017). 10.1111/tmi.12866 PMC10304464 28278357

[47] Sampson RJ Morenoff JD Gannon-Rowley T Assessing “neighborhood effects”: social processes and new directions in research. Annu Rev Sociol (2002) 28(1):443–78. 10.1146/annurev.soc.28.110601.141114

[48] Brooks-Gunn J Duncan GJ Klebanov PK Sealand N Do neighborhoods influence child and adolescent development? Am J Sociol (1993) 99(2):353–95. 10.1086/230268

[49] Duncan GJ Brooks-Gunn J Klebanov PK. Economic deprivation and early childhood development. Child Dev (1994) 65(2):296–318. 10.2307/1131385 7516849

[50] Fischer KW Bullock D Cognitive development in school-age children: conclusions and new directions. In: Collins WA, editor. Development during Middle Childhood: The Years from Six to Twelve. Washington, DC: National Academy of Sciences Press (1984). p. 70–146. 25032422

[51] Cadima J Gamelas AM McClelland M Peixoto C Associations between early family risk, children’s behavioral regulation, and academic achievement in Portugal. Early Educ Dev (2015) 26(5–6):708 10.1080/10409289.2015.1005729

[52] Bratsch-Hines ME Vernon-Feagans L The Family Life Project Key Investigators Child care changes, home environment quality, and the social competence of African American Children at age 3. Early Educ Dev (2013) 24:8 10.1080/10409289.2013.736359

[53] Grover VM Sebate KM Revised Manual for the Grover-Counter Scale of Cognitive Development. Pretoria: Human Sciences Research Council Library (2000).

[54] Hair JF Black WC Babin BJ Anderson RE Tatham RL Multivariate Data Analysis. 7th ed Upper Saddle River, NJ: Pearson Education (2009).

[55] Stevens J Applied Multivariate Statistics for the Social Sciences. 5th ed Mahwah, NJ: Lawrence Erlbaum (2009).

[56] Mistry RS Benner AD Biesanz JC Clark SL Howes C Family and social risk, and parental investments during the early childhood years as predictors of low-income children’s school readiness outcomes. Early Child Res Q (2010) 25(4):432–49. 10.1016/j.ecresq.2010.01.002

[57] Whittaker JEV Harden BJ See HM Meisch AD Westbrook TPR Family risks and protective factors: pathways to early head start toddlers’ social–emotional functioning. Early Child Res Q (2011) 26(1):74–86. 10.1016/j.ecresq.2010.04.007

[58] Rimm-Kaufman SE Curby TW Grimm KJ Brock LL Nathanson L The contribution of children’s self-regulation and classroom quality to children’s adaptive behaviours in the kindergarten classroom. Dev Psychol (2009) 45(4):958–72. 10.1037/a0015861 19586173

[59] Johnson AD Martin A Brooks-Gunn J Petrill SA Order in the house! Associations among household chaos, the home literacy environment, maternal reading ability, and children’s early reading. Merrill Palmer Q (2008) 54(4):445–72. 10.1353/mpq.0.0009 PMC2695402 19526070

[60] Petterson SM Albers AB. Effects of poverty and maternal depression on early child development. Child Dev (2001) 72(6):1794–813. 10.1111/1467-8624.00379 11768146

[61] Hooper D Coughlan J Mullen M Structural equation modelling: guidelines for determining model fit. Electron J Bus Res Methods (2007) 6(1):53–60.

[62] Klebanov PK Brooks-Gunn J Chase-Lansdale PL Gordon RA Are neighbourhood effects on young children mediated by features of the home environment? In: Brooks-Gunn J Duncan GJ Aber JL, editors. Neighborhood Poverty: Context and Consequences for Children. New York, NY: Russel Sage (1997):119–45.

[63] Joo M Long-term effects of head start on academic and school outcomes of children in persistent poverty: girls vs. boys. Child Youth Serv Rev (2010) 32(6):807–14. 10.1016/j.childyouth.2010.01.018

[64] Browning CR Cagney KA Neighborhood structural disadvantage, collective efficacy, and self-rated physical health in an urban setting. J Health Soc Behav (2002) 43(4):383–99. 10.2307/3090233 12664672

[65] Blau DM Hagy AP The demand for quality in child care. J Political Econ (1998) 106(1):104–46. 10.1086/250004

[66] Burchinal MR Peisner-Feinberg E Bryant DM Clifford R Children’s social and cognitive development and child-care quality: testing for differential associations related to poverty, gender, or ethnicity. Appl Dev Sci (2000) 4(3):149–65. 10.1207/S1532480XADS0403_4

[67] Love JM Harrison L Sagi-Schwartz A van Ijzendoorn MH Ross C Ungerer JA Child care quality matters: how conclusions may vary with context. Child Dev (2003) 74(4):1021–33. 10.1111/1467-8624.00584 12938696

[68] Ndukwu C Egbuonu I Ulasi T Ebenebe J. Determinants of undernutrition among primary school children residing in slum areas of a Nigerian city. Niger J Clin Pract (2013) 16(2):178–83. 10.4103/1119-3077.110142 23563458

[69] Martin P Child Food and Nutrition Security. PAN: Children (2012). Available from: http://children.pan.org.za/

[70] Said-Mohamed R Micklesfield LK Pettifor J Norris SA Has the prevalence of stunting in South African children changed in 40 years? A systematic review. BMC Public Health (2015) 15:534 10.1186/s12889-015-1844-9 26044500 PMC4456716

[71] South African Early Childhood Review. Children’s Institute, Department of Planning, Monitoring & Evaluation, Ilifa Labantwana (2016). Available from: http://www.bettercarenetwork.org/sites/default/files/South%20African%20Early%20Childhood%20Review%202016.pdf

[72] Statistics South Africa. Community Survey 2016, Statistical Release P0301. Pretoria: Statistics South Africa (2016).

[73] United Nations Children’s Fund (UNICEF). UNICEF’s Approach to Scaling Up Nutrition for Mothers and Their Children. Discussion Paper. New York, NY: Programme Division, UNICEF (2015).

[74] National Development Agency. Early Childhood Development (2017). Available from: http://www.nda.org.za/home/Early_childhood_development-23.html

[75] Burchinal MR Howes C Pianta R Bryant D Early D Clifford R Predicting child outcomes at the end of kindergarten from the quality of pre-kindergarten teacher–child interactions and instruction. Appl Dev Sci (2008) 12(3):140–53. 10.1080/10888690802199418

[76] Lichtenberger EO Volker MA Kaufman AS Kaufman NL Assessing gifted children with the Kaufman assessment battery for children-second edition (KABC-II). Gift Educ Int (2006) 21(23):99–126. 10.1177/026142940602100304

